# GECO: gene expression clustering optimization app for non-linear data visualization of patterns

**DOI:** 10.1186/s12859-020-03951-2

**Published:** 2021-01-25

**Authors:** A. N. Habowski, T. J. Habowski, M. L. Waterman

**Affiliations:** Department of Microbiology and Molecular Genetics, University of California Irvine, Irvine, CA 92697 USA

**Keywords:** RNA-seq, Gene expression, t-SNE, UMAP, PCA, Clustering, App, Data visualization

## Abstract

**Background:**

Due to continued advances in sequencing technology, the limitation in understanding biological systems through an “-omics” lens is no longer the generation of data, but the ability to analyze it. Importantly, much of this rich -omics data is publicly available waiting to be further investigated. Although many code-based pipelines exist, there is a lack of user-friendly and accessible applications that enable rapid analysis or visualization of data.

**Results:**

GECO (Gene Expression Clustering Optimization; http://www.theGECOapp.com) is a minimalistic GUI app that utilizes non-linear reduction techniques to rapidly visualize expression trends in many types of biological data matrices (such as bulk RNA-seq or proteomics). The required input is a data matrix with samples and any type of expression level of genes/protein/other with a unique ID. The output is an interactive t-SNE or UMAP analysis that clusters genes (or proteins/other unique IDs) based on their expression patterns across the multiple samples enabling visualization of expression trends. Customizable settings for dimensionality reduction, data normalization, along with visualization parameters including coloring and filters, ensure adaptability to a variety of user uploaded data.

**Conclusion:**

This local and cloud-hosted web browser app enables investigation of any -omic data matrix in a rapid and code-independent manner. With the continued growth of available -omic data, the ability to quickly evaluate a dataset, including specific genes of interest, is more important than ever. GECO is intended to supplement traditional statistical analysis methods and is particularly useful when visualizing clusters of genes with similar trajectories across many samples (ex: multiple cell types, time course, dose response). Users will be empowered to investigate -omic data with a new lens of visualization and analysis that has the potential to uncover genes of interest, cohorts of co-regulated genes programs, and previously undetected patterns of expression.

## Background

The next generation sequencing revolution has resulted in the production of an enormous amount of data [1, 2]. While much of this data is available in public repositories or supplementary manuscript material, there remains a bottleneck in a broader public analysis of the data. Thus, the ability to further our understanding of the world thru an -omics lens is limited not by the production of data, or even its accessibility, but by our ability to analyze it. Although others have developed pipelines to aid in re-analyzing publicly available data [3], it is important to develop analysis pipelines that enable quick, accessible, and easy use of already available data matrices to encourage their broad utilization. Currently, there are numerous bioinformatic pipelines to statistically analyze -omic data, however the majority are dependent on being able to run code, an expertise lacking for many biologists. Thus, there is a great need for GUI (Graphical User Interface) based programs that circumvent prerequisites for coding skills [4–6]. An easy-to-use data analysis tool which also facilitates data exploration, can lead to new insights. Importantly, since many publications are accompanied by already processed data matrices, a rapid and user-friendly method to analyze these data-matrices is informative and could broaden and deepen analysis of publicly available data.

Many classic differential expression analyses result in outputs of tables of genes with statistics, volcano plots, or heat maps showing strongly differentially expressed genes between samples [7–9]. Although these analyses are useful, they also make it difficult to visualize the data globally and identify cohorts of genes that might be behaving in a similar manner across samples. Identifying these cohorts of genes can lead to investigation of impacted gene programs or classes of ontology that might be overlooked when sorting through list of genes by significance alone. Additionally, many of the bulk RNA-seq pipelines for differentially expressed genes cater to paired analysis—generally between a control and experimental samples. This can make comparisons across a cohort of samples such as a dose–response curve, multiple genotypes, and/or time courses very challenging. Although there have been specialized pipelines for the analysis of time courses, in many cases these pipelines are still outperformed by pairwise analysis [10, 11]. There is a need for analyses that can visualize gene patterns and trends across all samples at the same time.

The increased quantity of sequencing data and the rise of single cell sequencing data has been reliant on more complex bioinformatic analyses, which has further encouraged a merge of the fields of computer science and biology [2, 12, 13]. Several unsupervised approaches have been borrowed from machine-learning such as PCA (Principal Component Analysis), t-SNE (t-distributed Stochastic Neighbor Embedding), and UMAP (Uniform Manifold Approximation and Projection). PCA is a mathematical approach that uses a linear dimensionality reduction method to investigate data relatedness [14, 15]. In essence, PCA reduces the data to eigenvectors showing how related data points are to one another. The dominant two principal components can usually separate data based on the largest variance. Although PCA can rapidly reduce complex data, visualizing highly dimensional data with PCA has limitations [14, 15]. Non-linear dimensionality reduction using probabilistic approaches, such as t-SNE [16] and UMAP [17], better enable visualization of complex-multidimensional data in a low dimensional space. Although these techniques were developed by computer scientists for machine learning applications, they have found a prominent home in analyzing the growing expanse of single cell -omic data [18, 19]. These non-linear dimensionality reduction techniques better preserve the complexity of the data and importantly, the closeness of data points can be used to draw conclusions on the relatedness between these points. Previous publications have shown the value and usefulness of non-linear over linear dimension reduction and the ability to customize and optimize the parameters [19–21].

Here we present GECO (Gene Expression Clustering Optimization), a minimalistic GUI app that utilizes non-linear reduction techniques to visualize expression trends in biological data matrices (such as bulk RNA-seq, single cell RNA-seq, or proteomics). The required input is a data matrix with samples and any type of expression level of genes/protein/other unique ID. The output is an interactive t-SNE or UMAP graphical representation that clusters genes (or proteins/unique IDs) based on expression patterns across samples to enable visualization of trends. Each data point on the plot is one gene/protein/other unique ID with the expression pattern across all samples used to determine its position and location relative to other data points. Features of GECO include:

- User-friendly Streamlit run app accessed through a cloud-hosted website (no code, downloading, or installation needed). (http://www.theGECOapp.com)
- Option to run Streamlit locally on user’s computer with network host capability for temporary sharing.
- Customizable parameters for t-SNE and UMAP generation (optional PCA initial reduction).
- Optional GPU driven clustering for t-SNA and UMAP generation.
- Save function for t-SNE and UMAP enabling re-opening of a saved interactive session (important for stochastic analysis like t-SNE and UMAP where each run will yield variation and a different cluster shape).
- Flexible data type input.
- Optional normalization techniques, filtering, and threshold cutoff.
- Incorporation of curated marker genes, gene searching, and highlighting function.
- Autogenerated bar plot, correlation clustermap (with significance calculated), and heatmap expression of selected genes.
- Generation of downloadable gene list based on clustering and filtering.
- Large selection of colors, inversion and log of scale functions, and.png generation of plots to facilitate user flexibility based on needs/preferences.

## Implementation

### Architecture

All code for GECO was written in Python 3.7 and uses Streamlit (https://www.streamlit.io/) and Plotly (https://plotly.com/) for GUI and interactive data visualization. Streamlit is a new open source app framework that was chosen for its relative simplicity to implement a graphical interface to the python back end code. All source code, install files, and install directions for GECO are available on github (https://github.com/starstorms9/geco; and Additional file 1). GECO is intended to be used without any programming knowledge. A cloud-hosted website version of GECO thru *Streamlit for Teams* (currently in beta form) can be accessed at http://www.theGECOapp.com. In order to run GECO locally, step-by-step installation instructions are available on github. README documentation is provided in Additional file 2, including step-by-step instructions for data analysis and utilization of optional features (also available on github).

### User interface

**Fig. 1:**
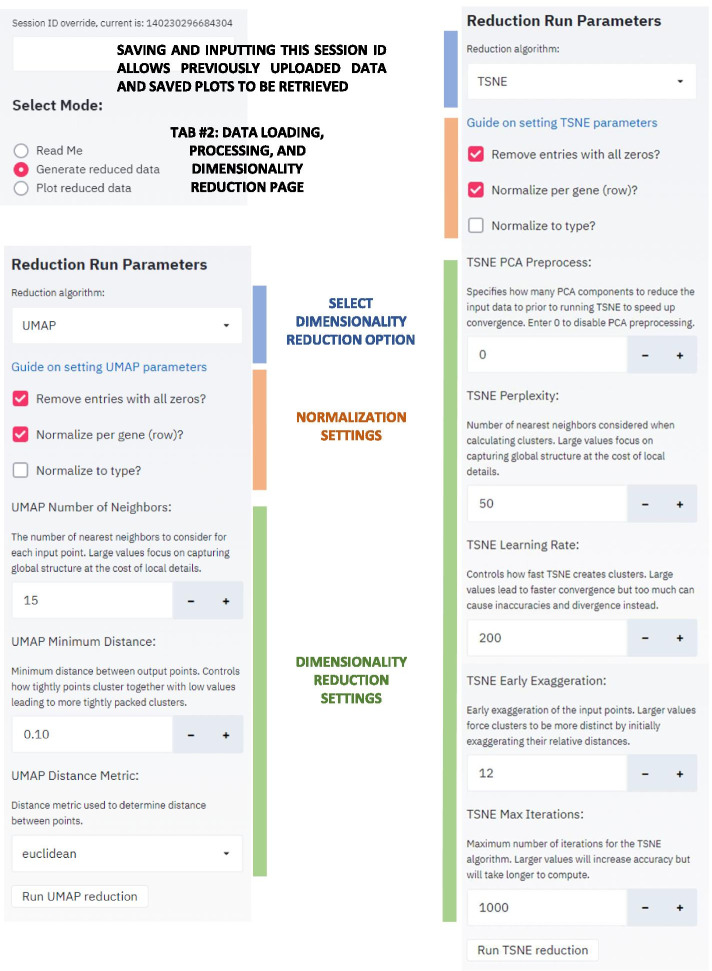
GECO app interface to generate reduced date. Once a data matrix is uploaded in the main window and samples are identified, this side bar is used to select the desired reduction type (t-SNE or UMAP). Normalization options include default settings of removing entries with all zeros and row normalization. Normalizing to a selected type is optional. Parameter options for t-SNE include initial PCA reduction, perplexity, learning rate, early exaggeration, and iteration number. UMAP parameters include number of neighbors, minimum distance, and distance metric. Standard default settings automatically appear, but links to t-SNE and UMAP parameter guides are provided to aid in exploration and customization

**Fig. 2:**
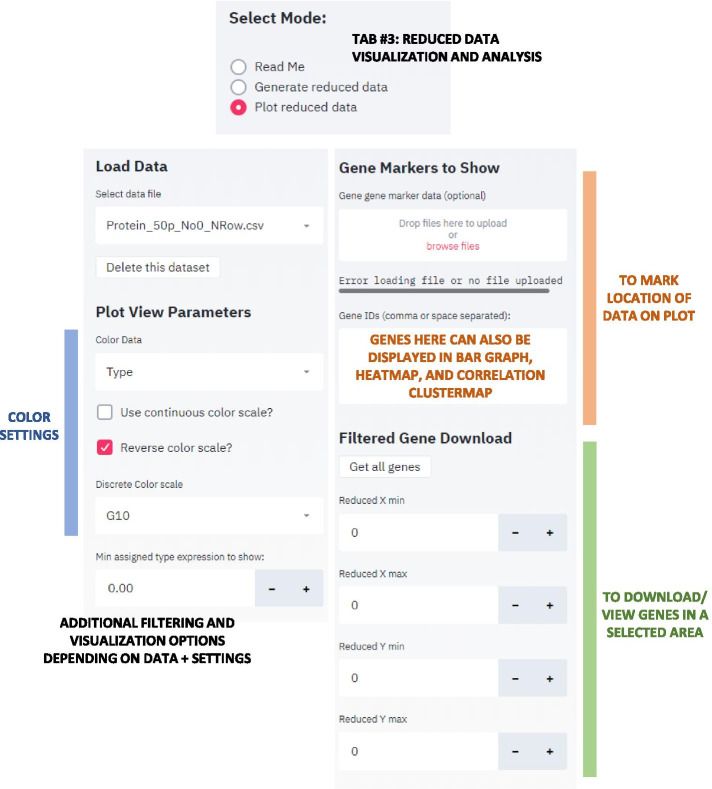
GECO app interface for plotting and investigating reduced data. This tab and interface enable investigation of a previously saved plot of reduced data. The data displayed and color settings can be adjusted and filtered. Optional gene marker lists can be uploaded or input in the Gene ID box to be highlighted on the plot or further investigated in bar graph, clustermap, or heatmap. A gene list from a region of interest can be generated using the coordinate system

**Fig. 3:**
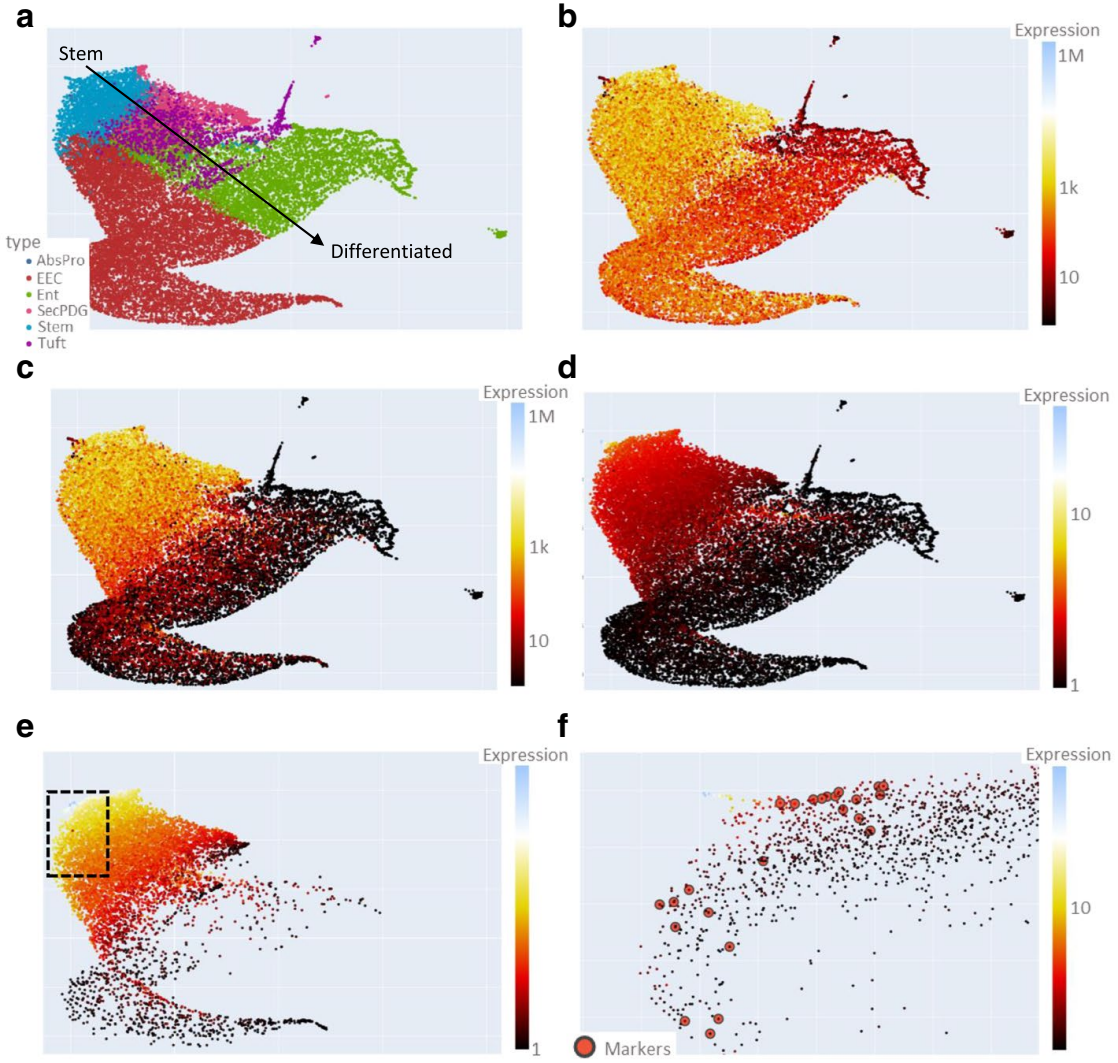
Investigation of intestinal stem genes using GECO. UMAP generated plot of colon crypt cell types with the following settings: row normalization, removal of zeros, number of neighbors = 35, minimum distance = 0.5, distance metric = Manhattan. Data points (genes) colored by **a** type, **b** average expression of assigned type, **c** stem expression, **d** stem enrichment, **e** stem enrichment with 500 (normalized counts) minimum expression level with box showing zoomed in region displayed in **f**. **f** Zoomed in region with stem enrichment coloring and a 1.5-fold cutoff. Highlighted in red circles are 27 genes that were previously identified as being statistically differentially expressed and enriched in stem cells in this dataset [23]. Previously published bulk RNA-seq of colon crypt cell types [23] was used to generate UMAP clustering and this dataset (.csv file) is available in Additional file 4

The Streamlit interface utilizes three main tabs: (1) a landing page that documents usage (Additional file 2: README), (2) a data loading, processing, and dimensionality reduction page (Fig. 1), and (3) a reduced data visualization page (Fig. 2). On each page, the sidebar provides access to the majority of the controllable parameters and the main screen shows the results. At the top of the sidebar interface is an assigned Session ID number which the user should save because uploading this ID number later allows the user to re-access the current session including the uploaded datasets and saved plots. Sharing the Session ID number is also an easy way to allow collaborators to explore shared datasets.

In the data visualization tab, the reduced dimensionality data can be investigated with a variety of customizable options. The visualization options were developed by investigating various datasets with known trends and features and exploring the ways to highlight these features most clearly. This strategy facilitated identification of interesting trends in new and unexplored datasets. For example, normalizing to a specific control type and selecting entries that have a high fold change relative to the control quickly highlights entries that were most affected by a given condition. The visualization tab also allows a user to select entries of interest and generate a bar graph, a correlation clustermap, and a heatmap to compare the subset of entries to each other.

### Data input and output

In order to readily accommodate a wide variety of input data from disparate sources, GECO has a system for automatic data cleaning to ensure that the data loaded into the dimensionality reduction algorithms are properly formatted. It is important to note that GECO does not perform any statistical analysis or filter for statistical significance. If this is important for the user’s analysis, preprocessing and filtering of the dataset prior to uploading is recommended. During testing, any issues encountered with loading test datasets were used to develop automatic solutions. For example, it was found that many datasets contain a significant quantity of entries with all 0′s or entries with some non-numeric characters, entries that can distort the output of the processing algorithms. GECO provides simple options to remove these confounding entries. Additionally, because naming conventions of samples and bio reps is highly varied, a system was implemented to recognize and group similarly named samples into coherently labeled sets.

After the data has been uploaded and processed through dimensionality reduction algorithms, the post-processed data can be saved and then visualized. Options are also available to manually enter or upload a comma separated list of entries of particular interest which are then marked prominently on the plot. Once specific groups of interest have been identified, they can then be downloaded along with their relevant reduced dimensionality parameters for further analysis externally.

### Algorithms

Three core algorithms were implemented for dimensionality reduction: PCA, t-SNE, and UMAP. Existing implementations of these algorithms were available as open source python modules. Due to the generally long processing time and high degree of parallelization possible with the t-SNE algorithm in particular, a CUDA based implementation called t-SNE-CUDA [22] was used for GECO. This t-SNE implementation is approximately ~ 50 times faster than standard CPU based algorithms and allows for rapid exploration of the effects that various hyperparameters such as perplexity and learning rate have on the final output. However, GPU enabled implementations of t-SNE are currently only available on Linux based systems and so a backup CPU based implementation is automatically utilized when the program is run on other systems. PCA analysis alone was insufficient to visualize the data clearly but it is used as a preprocessor before the t-SNE algorithm runs in order to reduce the number of variables and make the calculation time for the t-SNE tenable. UMAP is another popular non-linear reduction technique and is implemented here as it captures global correlations and structure more accurately compared to t-SNE which primarily focuses on local structure. UMAP performance speed also far outperforms t-SNE (when run without t-SNE-CUDA) and is recommended for faster dimensionality reduction times.

There are two important normalization options that can be applied to the data before running the dimensionality reduction algorithm: (1) normalize per row and/or (2) normalize to type. To normalize per row, every entry is scaled down by the sum of that row. This strategy ensures that the algorithm focuses only on the relative pattern for a given entry instead of just the overall magnitude of that entry. When no row normalization is performed for gene expression data the resulting reduced dimensionality plots are often simply aligned according to the overall expression levels and ignore more interesting, but subtle expression patterns that are shared by genes that are expressed in similar ways (Additional file 3: Fig. S1). Likewise, normalizing every entry to a selected type (e.g. control) prior to reducing the data ensures that the reduction algorithm focuses on the patterns that change relative to the control instead of looking at global patterns.

## Results and discussion

### Example usage 1: colon crypt cell types (bulk RNA-seq)

The inner layer of the colon contains epithelial cells in a crypt structure including proliferating stem cells. These stem cells give rise to immature daughter cells which then further differentiate into mature cells. Previously, bulk RNA-seq was performed on sorted epithelial crypt populations including stem cells, immediate daughter cells (AbsPro, SecPDG), and more mature differentiated cells (Tuft, Ent, and EEC) (Additional file 4) [23]. A UMAP plot generated with GECO from this dataset and colored by assigned cell type shows a gene expression trajectory of stem related genes which transition to those associated with more differentiated cell types (Fig. 3a). All cell types have assigned genes that are strongly expressed (Fig. 3b). Coloring the data points (genes) by expression in stem cells reveals the clustering of highly expressed stem-associated genes to one region (Fig. 3c). Further, coloring by stem enrichment shows a smaller region where the genes are highly expressed in stem, and less expression in other cell types (Fig. 3d). Filtering the genes displayed on the plot by removing those genes that have an expression below a minimum (500 normalized counts) further highlights the cohort of genes enriched in stem cells (Fig. 3e). Zooming in on a region of interest and adding a filter for a 1.5-fold cutoff for stem enrichment reveals clustering of stem-associated genes (Fig. 3f). As a comparative, validation analysis, we displayed stem cell marker genes (n = 27) that have been previously identified using traditional statistical differential expression methods (DESeq2), and they are clustered in this region as well [23].

In this example, GECO enables visualization of genes that are enriched in different cell types in the colon crypt. The clustering of genes assigned to each cell type follow the natural trajectory of stem cells to daughter cells to differentiated cells. Previously identified stem markers overlap well with the genes that survive the filtering cutoff of enriched expression in stem cells. Although the GECO plot in Fig. 3f displays many genes of potential interest, additional stringent filtering could also be applied to decrease the data points. However, in this case GECO is able to rapidly reveal the trajectory of gene expression changes in these cell types and identify stem-associated genes.

### Example usage 2: infection time course of *F. nucleatum* (bulk RNA-seq)

**Fig. 4:**
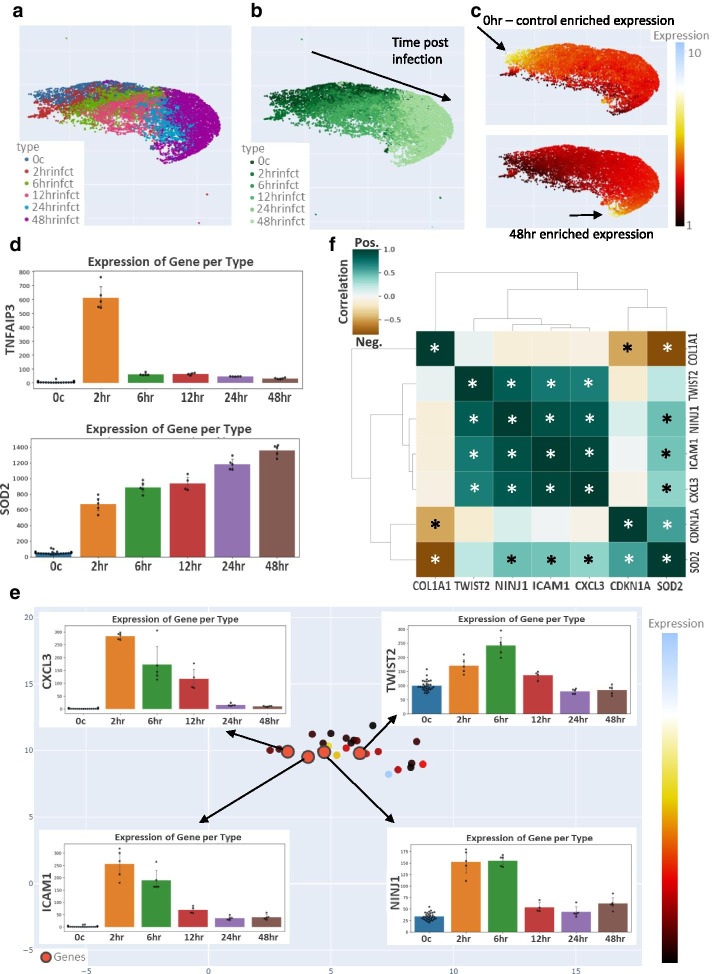
Gene expression patterns during infection time course. UMAP generated plot of *F. nucleatum* infection at time points 0, 2, 6, 12, 24, and 48 hrs with the following settings: row normalization, removal of zeros, number of neighbors = 15, minimum distance = 0.10, distance metric = Cosine, minimum expression = 1. Data points (genes) colored by **a** type, **b** type with continuous color setting, and expression of enrichment in **c** 0 h and 48 h. **d** Selected genes of interest graphed using GECO. **e** Data points (genes) colored by expression of enrichment in 6 h with minimum expression = 150 and 1.5-fold cutoff filter (See Additional file 3: Fig. S2a for UMAPs of addition step-by-step changes in filtering). Small subset of remaining genes following filtering with 4 genes highlighted with a red dot (*TWIST2*, *ICAM1*, *CXCL3*, and *NINJ1*) that are enriched in 6 h and 2 h infection time points. **f** Correlation of *F. nucleatum* infection related genes displayed in a GECO generated clustermap. Dark teal boxes with an asterisk are significantly positively correlated in expression across all samples (ex: *ICAM1* and *CXCL3*), whereas dark brown boxes with an asterisk are anti-correlating across all samples (ex: *COL1A1* and *SOD2*). Previously published bulk RNA-seq of *F. nucleatum* infection time course [24] was used to generate UMAP clustering and this dataset (.csv file) is available in Additional file 5

*Fusobacterium nucleatum* (*F. nucleatum*) is a pathogen that frequently contributes to periodontal diseases. Previous work investigated the impact of *F. nucleatum* infection on human gingival fibroblasts using a time course of bulk RNA-seq (Additional file 5) [24]. A GECO generated UMAP plot colored by assigned type (Fig. 4a) reveals the genes are clustered tightly based on expression at different time points during injection (0, 2, 6, 12, 24, and 48 h). This is further evident when the continuous color setting is used and a clear trajectory from 0 h (control) to 48 h post infection emerges (Fig. 4b). Altering the color setting to expression fold enrichment for 0 h versus 48 h (Fig. 4c) highlights the genes that are most highly expressed at 0 h (top left corner of the plot), compared to genes that are more highly expressed at 48hrs (bottom right corner of the plot). Cohorts of genes can be identified that are enriched at specific time points or that gradually increase or decrease over the duration of the infection. Figure 4d (and Additional file 3: Fig. S2a) shows several examples of selected genes graphed using GECO where each data point was a bio-replicate from the uploaded dataset.

To identify a small cohort of genes elevated early during *F. nucleatum* infection, the UMAP plot was colored based on enrichment at 6 h and then restricted to a minimum 1.5-fold cutoff or greater (Additional file 3: Fig. S2b). This highlights a small region of genes that are elevated in the first several hours of infection compared to all other time points. This selection was further filtered to find ~ 25 highly expressed genes (minimum expression cutoff of 150) (Fig. 4e). This gene list was then printed to the screen and four genes with trends of interest are marked with red circles on the UMAP plot and displayed in GECO-generated bar graphs. The gene list includes *CXCL3* and *ICAM1* which are sharply induced at 2 h from the start of infection and then gradually decline, *TWIST2* which peaks at 6 h, and *NINJ1* which is elevated at 2 and 6 h (Fig. 4e and GECO generated heatmap in Additional file 3: Fig. S2c). Displaying these genes, along with those displayed in Fig. 4d and Additional file 3: Fig. S2a, in a GECO generated correlation clustermap shows a significant correlation between this cohort of 4 genes (*CXCL3, ICAM1, TWIST2,* and *NINJ1)* (Fig. 4f). *COL1A1* and *SOD2* show inverse trends and as expected the clustermap reveals they are significantly anti-correlated (Fig. 4f). Previously published work using standard differential expression analysis identified 22 genes that were significantly upregulated throughout *F. nucleatum* infection, including SOD2, CXCL3, and ICAM1 [24]. Our analysis here confirms elevated expression of SOD2 (Fig. 4d), but suggests that CXCL3 and ICAM1, despite being significantly upregulated, follow a different pattern. CXCL3 and ICAM1 instead had a burst in expression at 2 h, and then gradually decrease (Fig. 4e). This comparative data analysis highlights the usefulness of GECO in uncovering gene expression patterns that could be overlooked by tradition differential expression analysis but may reveal important biology.

GECO is useful to visualize gene expression changes across multiple samples such as a time course and can be used to define cohorts of genes with matching gene expression trends. In this dataset there is a clear trajectory of genes that are elevated in the 0 h-control samples or at each time point (ex: *TNFAIP3*), whereas other genes gradually change over the time course and peak at one datapoint (ex: *COL1A1* and *SOD2*). In the latter case, these are often genes that are difficult to uncover with traditional statistical differential expression analysis (particularly paired analysis), but when looking at global trends such as those that GECO enables, these genes can be uncovered along with other genes that behave in a similar pattern.

### Example usage 3: pancreatic cancer metastasis (single cell RNA-seq)

GECO can also be used to investigate gene expression patterns in single cell RNA-seq data, but it functions with some constraints. For example, we investigated publicly available Fluidigm data (limited number of cells) collected from a primary pancreatic ductal adenocarcinoma (PDAC), liver metastasis, and circulating tumor cells from a highly metastatic patient-derived xenograft model [25]. GECO treats each cell as a bio-replicate sample of the tissue of origin (primary tumor, circulating tumor cell, or liver metastasis), and clusters genes based on expression across these groups (Additional file 3: Fig. S3a, c). Additionally, single cells could also be grouped based on identified clusters (i.e. after using Seurat [26]) to investigate global expression trend differences amongst clusters. Following a transformation of the data (flipping X and Y axis), GECO is able to plot a limited number of single cells, rather than genes, and expression of genes of interest can be further investigated (Additional file 3: Fig. S3b, c). Although designed and optimized for bulk RNA-seq, the ability to also analyze some single cell datasets highlights the ingenuity and multi-purpose functions of GECO.

## Conclusion

GECO is a minimalistic Streamlit GUI app that utilizes non-linear reduction techniques to visualize expression trends in biological data matrices. This app enables investigation of any -omic data matrix in a rapid and code-independent manner. With the continued growth of available -omic data, the ability to quickly evaluate a dataset, including specific genes of interest, is more important than ever. GECO is intended to supplement more traditional statistical analysis methods and is particularly useful when visualizing clusters of genes with similar trajectory across many samples (ex: multiple cell types, time course, dose response). With a variety of options for dimensionality reduction, normalization methods, and visualization (coloring), along with thorough step-by-step instructions, users will be empowered to investigate their -omic data with a new lens with the potential to uncover genes of interest and previously unseen patterns.

## Availability and requirements

- **Project name**: GECO.
- **Project home page**: http://www.theGECOapp.com & https://github.com/starstorms9/geco
- **Operating system(s)**: Linux, Windows, Mac.
- **Programming language**: Python 3.7 + 
- **Other requirements**: Streamlit, Plotly, Scipy, Pandas, Seaborn, Umap-Learn, t-SNE-CUDA, numpy.
- **License**: MIT License.
- **Any restrictions to use by non-academics**: None.

## Supplementary Information

**Additional file 1:** GECO python source code.

**Additional file 2:** GECO README documentation and step-by-step instructions.

**Additional file 3:** Figures S1–S3.

**Additional file 4:** CSV file of colon crypt bulk RNA-seq data used for GECO UMAP generation.

**Additional file 5:** CSV file of bulk RNA-seq data of *F. nucleatum* infection time course used for GECO UMAP generation.

## Data Availability

The datasets used/analyzed as part of this study are previously published [23–25] and are also available in Additional files 4 and 5.

## Abbreviations

GECO: Gene Expression Clustering Optimization
GUI: Graphical User Interface
PCA: Principal Component Analysis
t-SNE: T-distributed Stochastic Neighbor Embedding
UMAP: Uniform Manifold Approximation and Projection
RNA-seq: RNA sequencing
