# Supplementary material for: GECO: gene expression clustering optimization app for non-linear data visualization of patterns
Source: BMC Bioinformatics. 2021 Jan 25;22:29. doi: 10.1186/s12859-020-03951-2 (PMC7831185; doi:10.1186/s12859-020-03951-2)
Supplement: Supplementary file 2 — Additional file 2: GECO README documentation and step-by-step instructions. [file 12859_2020_3951_MOESM2_ESM.docx]

**GECO – README** *www.TheGECOapp.com*

Welcome to GECO (Gene Expression Clustering Optimization), the straightforward, user friendly [Streamlit app](https://www.streamlit.io/) to visualize and investigate data patterns with non-linear reduced dimensionality plots. Although developed for bulk RNA-seq data, GECO can be used to analyze any .csv data matrix with sample names (columns) and type (rows) [type = genes, protein, any other unique ID]. The output is an interactable and customizable t-SNE/UMAP analysis and visualization tool. The visualization is intended to supplement more traditional statistical differential analysis pipelines (for example DESeq2 for bulk RNA-seq) and to confirm and/or reveal new patterns.

If questions or issues arise please contact Amber Habowski at [Habowski@uci.edu](mailto:Habowski@uci.edu) or post an issue on the github issues page [here](https://github.com/starstorms9/geco/issues).

[GECO Video demonstration in 3 minutes](https://youtu.be/wo8OW7eiJ5k)

**Quick Guide to Getting Started ------------------------------------------------------------------------------------**

- - - 1. Upload data file and verify that GECO has interpreted the sample names/bio-replicates.
      2. Select the reduction parameters to be used for the analysis.
      3. Click the 'Run UMAP/t-SNE reduction' button at the bottom of the parameters sidebar.
      4. Once a plot is generated, save it by clicking ‘Save data file’ at the bottom of the sidebar.
      5. Proceed to the ‘Plot reduced data’ mode to visualize the saved plot.

**File Upload ---------------------------------------------------------------------------------------------------------------**

*(required)* The Data Matrix:

- Must be supplied as a .csv file.
- The first column should contain the unique IDs for this dataset (genes, isoforms, protein, or any other identifier) which will be renamed ‘geneid’ in the program. Each unique ID should have ‘expression’ data listed in each row that corresponds to each sample.
- Sample names must be listed at the top of each columns, with biological replicates being indicated by ‘_#’ following each sample name. Biological replicates are averaged during the analysis and the number of biological replicates does not need to match between samples. For example, for two samples (‘Asample’ and ‘Bsample’) with three biological replicates each the column names should be assigned as shown in the example below.
  - If no ‘_#’ columns are found for a given sample name, but there are duplicated column names, they will automatically have sample numbers appended.
- The file should not have any index column (1,2,3,4) or other columns with additional information.
- 'NA' entries will be interpreted as unknowns and those entire rows will be removed
  - Also any of: ['-1.#IND', '1.#QNAN', '1.#IND', '-1.#QNAN', '#N/A N/A', '#N/A', 'N/A', 'n/a', 'NA', '', '#NA', 'NULL', 'null', 'NaN', '-NaN', 'nan', '-nan', '']
  -
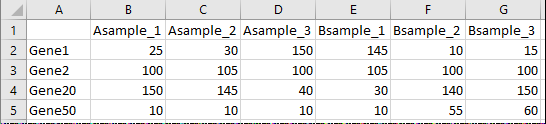
If there is a need to include rows with non-numeric values, the NA values must be imputed manually and replaced. For example, this can be achieved by “zero-ing” non-numeric values ­, replacing NA values with average of samples, etc.

*(optional)* Curated Markers List.

- Must be supplied as a .csv file.
- Each column should start with a descriptive title (that will appear in a drop-down list). Below the title will be a list of unique IDs (that overlap with the provided data matrix). A minimal example is provided below.
- Multiple curated lists can be provided by listing them next to each other, one per column. Do not skip columns, and do not use different excel sheet tabs.
-
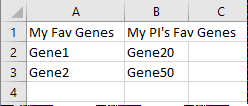
GECO is case sensitive, so make sure capitalization of gene entry text is consistent with the provided Data Matrix.

**Usage Instructions** **-----------------------------------------------------------------------------------------------------**

***Session ID Information:***

At the top of the sidebar is information on the current session ID and a box to input a previously saved session ID. This enables a previous session (including uploaded data and saved plots) to be reloaded for further investigation. This is also a great way to share saved analysis with collaborators if using the cloud-hosted version of GECO.

***Generate reduced dimensionality data:***

1. Start with the ‘Generate reduced data’ mode (click the ‘>’ in the top left to reveal the sidebar menu with Select Mode option.)
2. Upload data matrix in the format described above (“File Upload”). Review the inferred sample information from the upload – if the information is not correct, update the .csv file accordingly.
3. Set Reduction Run Parameters:
   1. See info [here](https://distill.pub/2016/misread-tsne/) for setting t-SNE parameters
   2. See info [here](https://pair-code.github.io/understanding-umap/) for setting UMAP parameters
   3. Three optional boxes can be checked:
      1. Remove entries with all zeros
      2. Normalize per gene (row) – this function divides each entry of a given row by the sum of the entire row and thus allows for investigation of trends across samples *independent* of overall expression level.
      3. Normalize to type– This function normalizes each row to a specified sample type (a drop-down menu will allow you to select a type for normalization) and allows for investigation of trends relating to the fold change compared to the selected type.
4. Run the reduction. This could take a bit of time depending on the size of the data. Active analysis is visualized by a ‘running’ icon in the top right corner of the app.
5. Preliminary data and manipulation are available. Enter a filename and save the data.
   1. Note that using the same filename as one that already exists will overwrite the file.

***Visualize the data:***

1. Switch to ‘Plot reduced data’ mode
2. Select the desired saved dataset from the drop-down list under ‘Load Data’.
3. Each dot/data point on the t-SNE/UMAP corresponds to one gene/protein/other unique ID depending on the input data. Using the ‘Color Data’ pull down menu the coloring of these data points can be altered in several ways:
   1. Expression of s*ample name/type* = Average expression/value based on each sample type – one at a time.
   2. Assigned type =Each data point is assigned the color of the sample type that has the highest expression/value for that gene/protein/other unique ID.
   3. Average expression of assigned type = The color scale is set to a range of expression values and each data point is colored for the average of the sample type that has the highest expression.
   4. Enrichment in type (select) = The color scale is set to a range of fold change of the selected sample (chosen in the dropdown) over maximum sample type expression. The color corresponding to the highest expression value will mark data points that are highest in the selected sample relative to other sample types.
4. Additional color/display options:
   1. Log Scale [only for some color data options] - transforms the scale of colors and is useful if there are prominent outliers overshadowing other data points.
   2. The Continuous Color Scale option [only for some color data options] - uses a continuous color gradient across discrete types to show transitions from one sample to the next. This analysis mode is particularly useful for time course or drug treatment when sample types are related.
   3. Reverse Color Scale switches the order of the colors.
   4. Sequential/Discrete Color Scales changes the overall colors used.
5. Additional filtering steps:
   1. Min expression of assigned type to show = removes data points with low expression values based on the number in the filter. Filters are based on the ‘assigned type’ which is the sample with the maximum expression.
   2. Min expression of *selected type* to show [only available for “Expression of *sample name/type”* color option] = removes data points below a specified threshold for the sample type selected to colorize the data.
   3. Fold change of selected type over average of other types [only available for the “Enrichment in type (select)” color option] = removes data points below a specified cut-off between selected type and other types.
6. ‘Gene Markers to Show’ will highlight the specified genes/proteins/unique IDs.
   1. Curated marker lists can be uploaded as a .csv file (genes in a column with descriptive header – files with multiple columns are accepted; see description in file upload format). The descriptive header will be used to select which marker gene list should be displayed. These lists will be highlighted on the plot with an ID dot (which is a large dot with a black outline).
   2. The Gene IDs input list will also be highlighted with an ID dot (the ID dot color will be different than the Curated list ID dots, if present).
   3. This is case sensitive and must match the given dataset – if a gene/protein/unique ID is not found it will not be displayed (no warning/error message), but other matching IDs from the list will be displayed.
   4. The ID dot will be a large circle appearing on the plot behind the data point. Depending on the color scale and density of data points, adjustments may need to be made to see the ID dot (filtering, zooming in, etc.). A key ‘Genes’ and/or ‘Markers’ will appear on the bottom left corner of the plot. By clicking on either of these terms, you will hide the circles while preserving the gene list.
7. Once at least one gene has been correctly entered in the Gene ID box, a bar graph will appear below the t-SNE/UMAP and plot the gene across the samples. If more than one gene has been entered the one displayed in the bar graph can be changed using the drop down ‘Gene to bar plot’ menu.
8. Once at least two genes have been correctly entered in the Gene ID box, two additional displays will appear:
   1. A clustermap showing the correlations of the selected genes. This displays all of the genes and calculates a correlation coefficient (significance of correlation or anti-correlation is shown with an asterisk).
   2. An expression heatmap of the specified genes across all samples. This is by default normalized by gene but the box that appears can be un-checked to disable this.
9. Under the ‘Filtered Gene Download’ there is a ‘Get all genes’ button that will print a list of all of the genes/proteins/unique IDs in the dataset. To print a specified cluster of genes type in the window of x and y coordinates and all genes from that specific range will be printed (with any plotting filters applied). Once the gene list is printed to the screen it can be downloaded (add the correct .csv extension to the end of the file name before opening).
10. The gene displayed can be adjusted by filtering as specified earlier or by zooming in/out on the plot. Zooming in can be performed by highlighting an area or hovering over the plot until a set of buttons appears in the top right corner (including +/- buttons). Double clicking on the plot returns to the default view. Hovering over a data point will display the gene and information on the expression in samples and the current color scale information.

**Troubleshooting/FAQ ------------------------------------------------------------------------------------------------**

1. File uploader utility says ‘files are not allowed’
   - Check that the file ends in a .csv and is a simple comma separated table.
2. Odd persistent issues with the app
   - You can soft reboot the app by hitting ‘c’ and ‘clear cache’ and then hit ‘r’ to reload.
3. The normalization options for ‘Normalize per gene (row)’ and ‘Normalize to type’ are not appearing as options when generating reduced data.
   - These two options are only available when more than two sample types are included in the data matrix. When these normalizations are used on only two sample types it results in a useless analysis.
4. TSNE takes a long time to run when using GECO locally, how can I make it faster?
   - To reduce the runtime of the t-SNE algorithm a GPU can be used. This requires a CUDA enabled graphics card (most Nvidia GPU’s), a Linux based system, and a more complex installation. However, using a GPU will reduce the t-SNE runtime down to only a few seconds for even very large datasets.
5. Given the same settings and dataset, why do each generated t-SNE and UMAP not look the same?
   - Both UMAP and t-SNE are stochastic reductions which means there is a level of randomness to where datapoints initially fall into the plotted space, however the relationships and trends between datapoints will be consistent.
6. What is the best way to save GECO generated data?
   - It is recommended to save the current session ID number as the cloud hosted web browser page can get reloaded/cleared and it does eventually time-out after a long duration of use.
   - Plots can also be downloaded as a .png to a local folder using the camera capture button just above the top right side of the plot (hover the mouse over the plot if these buttons are not visible).
   - Once a gene list is generated a link will appear enabling the full list to be generated. Once this download occurs add the necessary ‘.csv’ extension to the file name before opening.
7. What correlation metric is used for the clustermap?
   - A Pearson r correlation test is used. More info [here](https://en.wikipedia.org/wiki/Pearson_correlation_coefficient).
8. What are some suggested color scales?

- Blackbody
- Electric
- Jet
- Thermal
