# Supplementary material for: GECO: gene expression clustering optimization app for non-linear data visualization of patterns
Source: BMC Bioinformatics. 2021 Jan 25;22:29. doi: 10.1186/s12859-020-03951-2 (PMC7831185; doi:10.1186/s12859-020-03951-2)
Supplement: Supplementary file 3 — Additional file 3: Figures S1–S3. [file 12859_2020_3951_MOESM3_ESM.docx]

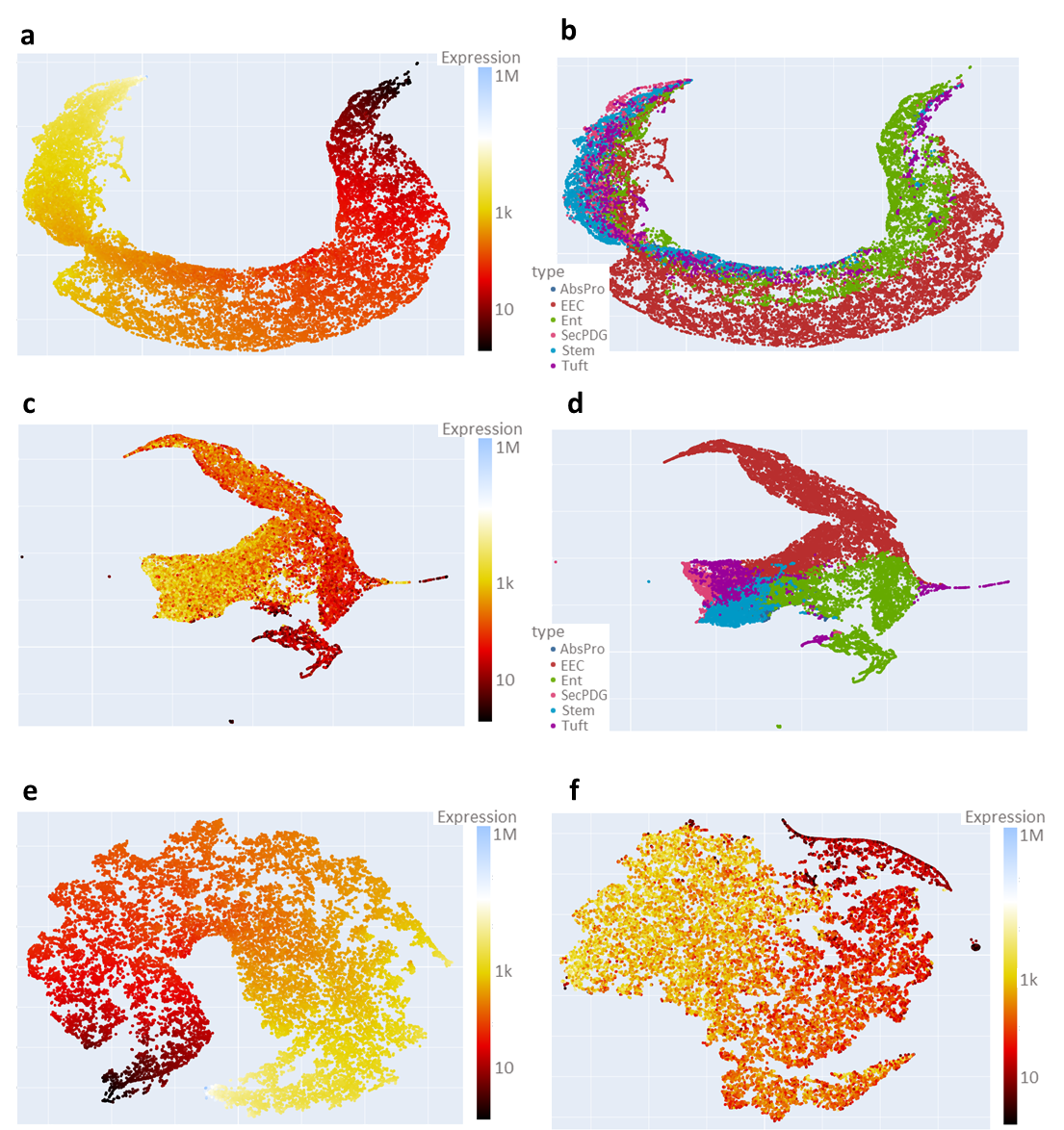


**Supplementary Figure 1:** *Data normalization influences clustering and usefulness of UMAP and t-SNE.*

UMAP generated without row normalization (removal of zeros, number of neighbors = 15; minimum distance = 0.10; distance metric = Euclidean) colored by **a** average expression of assigned type or **b** assigned type. Assigned type is the sample which has the highest expression of a given gene. UMAP generated with the same parameters in addition to row normalization colored by **c** average expression of assigned type or **d** assigned type. t-SNE generated plot (PCA = 0; perplexity = 50; learning rate = 200; early exaggeration = 12; max iterations = 1000) **e** without row normalization and **f** with row normalization. Previously published bulk RNA-seq of colon crypt cell types ^1^ was used to generate UMAP and t-SNE clustering and this dataset (.csv file) is available in Additional file 4.


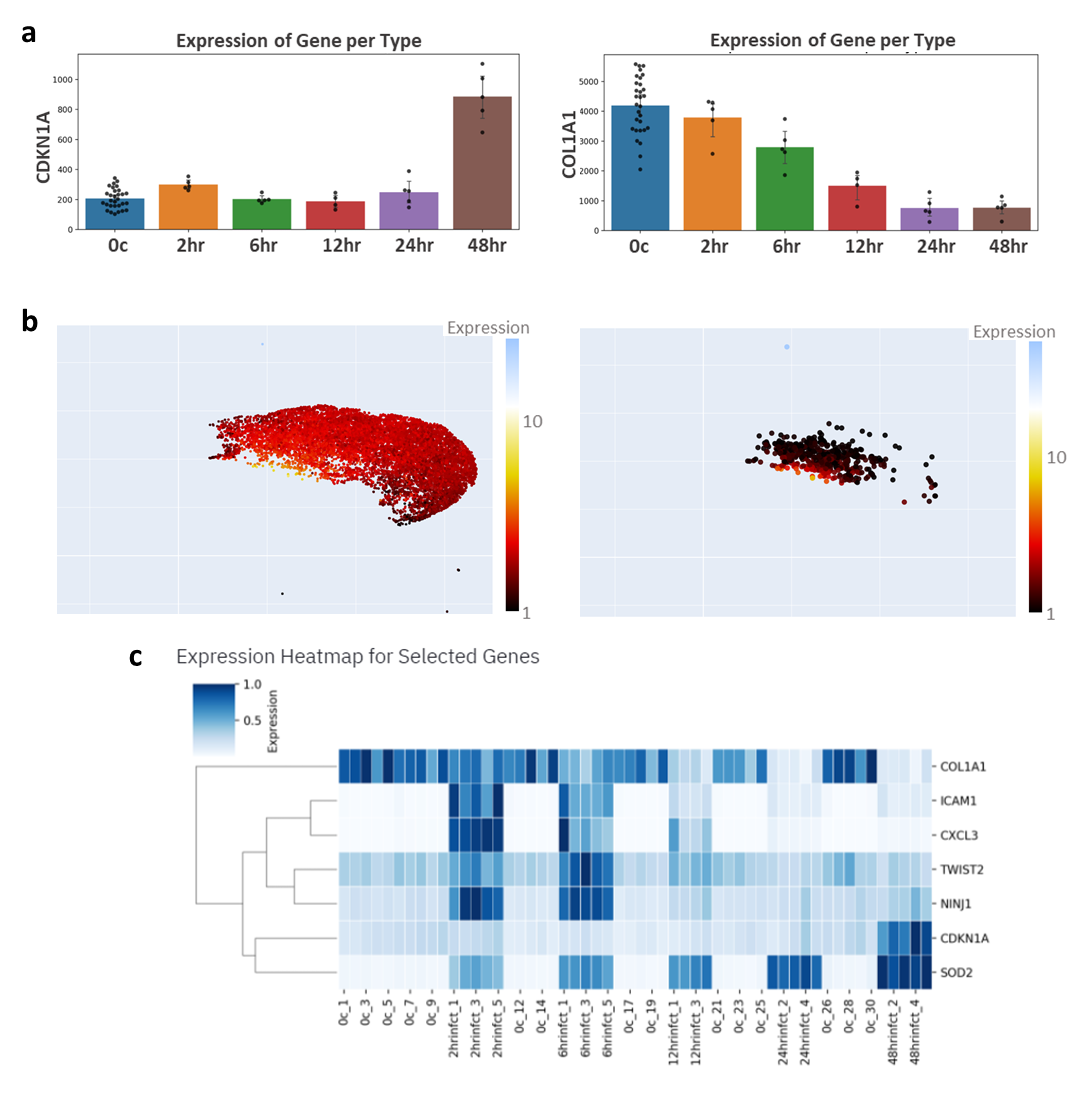


**Supplementary Figure 2:** *Identification of genes of interest during an infection using GECO.*

**a** Selected genes of interest in *F. nucleatum* infection at time points 0, 2, 6, 12, 24, and 48 hrs ^2^ graphed using GECO. **b** UMAP generated plot of *F. nucleatum* infection with the following settings: row normalization, removal of zeros, number of neighbors = 15, minimum distance = 0.10, distance metric = Cosine. Data points (genes) colored by expression of enrichment in 6 h with minimum expression = 1 (Left), and then with an additional 1.5-fold cutoff filter (Right). **c** GECO enables further visualization of genes of interest using heatmaps. Expression of selected genes in a GECO generated heatmap. Previously published bulk RNA-seq of *F. nucleatum* infection time course ^2^ was used to generate UMAP clustering and this dataset (.csv file) is available in Additional file 5.


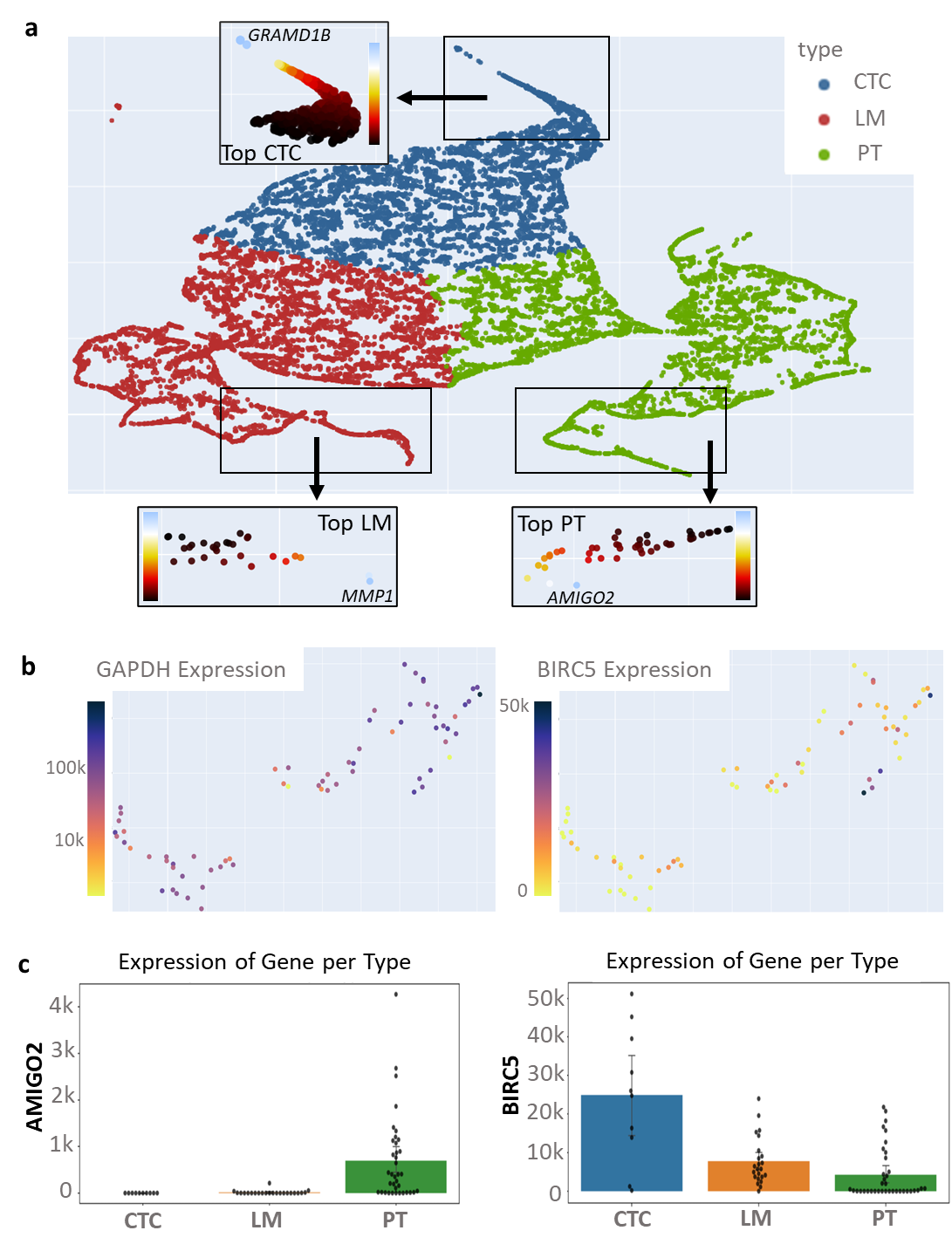


**Supplementary Figure 3:** *Single Cell RNA-seq analysis with GECO.*

Single cell RNA-seq (Fluidigm) data of pancreatic cancer ductal adenocarcinoma (PDAC) primary tumor (PT), liver metastasis (LM), and circulating tumor cells (CTC), was investigated using GECO ^3^. **a** UMAP generated plot three sample types (PT, LM, CTC) where each single cell was treated as a biological replicate. UMAP settings: row normalization, removal of zeros, number of neighbors = 35, minimum distance = 0.10, distance metric = Euclidean. Inset UMAP plots show the top genes colored by enrichment in either PT, LM, or CTC. Minimum expression of 500 counts and fold-change >5. A top enriched gene for each sample type is shown (*GRAMD1B, MMP1,* and *AMIGO2*). **b** Following transformation of the data matrix (swapping X and Y), GECO can be used to plot single cells, rather than genes. Single cells from PT, LM, and CTC (n=70) are plotted with UMAP settings: row normalization, removal of zeros, number of neighbors = 5, minimum distance = 0.0, distance metric = Euclidean. Cells are colored based on expression of housekeeping genes *GAPDH* and *BIRC5*, which was previously found to be enriched in CTCs and during metastasis ^3^. **c** GECO generated bar plots graph expression of single cells in three samples (CTC, LM, PT) and confirm elevated expression of *AMIGO2* in PT (as identified in **a**) and increase in *BIRC5* in CTC (as discussed in **b**).

**REFERENCES**

1. Habowski, A. N. *et al.* Transcriptomic and proteomic signatures of stemness and differentiation in the colon crypt. *Commun. Biol.* **3**, 1–17 (2020).

2. Kang, W. *et al.* Time-Course Transcriptome Analysis for Drug Repositioning in Fusobacterium nucleatum-Infected Human Gingival Fibroblasts. *Front. Cell Dev. Biol.* **7**, 204 (2019).

3. Dimitrov-Markov, S. *et al.* Discovery of New Targets to Control Metastasis in Pancreatic Cancer by Single-cell Transcriptomics Analysis of Circulating Tumor Cells. *Mol. Cancer Ther.* **19**, 1751–1760 (2020).
